# Democratic and Non-Democratic Elections

**Published:** 1918-05-18

**Authors:** 


### THE MATRONS' AND SISTERS' DEPARTMENT.

### DEMOCRATIC AND NON-DEMOCRATIC ELECTIONS.

The elections now proceeding for the College of Nursing Council are a matter of interest in circles far wider than that of the voters. The principle they embody, that of open nomination, is one which so far as we know has never been tried before in this country on so large a scale. The essential weakness of ordinary elections for the council or committees of societies lies in the nomination system. It is practically impossible for voters to reject the names submitted to them, or substitute others with any chance of success. The voting-papers of the Royal British Nurses' Association, for instance, now in the hands of its members, present a list of thirty persons willing to serve on the Council either as medical men, as matrons and superintendents of nurses, or as sisters and nurses, and this list has perforce been prepared by the executive committee. Members may, of course, strike out any name and substitute another. But this privilege is an empty one for this reason, that the names and addresses of the other members are only accessible to the secretary, so that combination is impracticable and no attempt to reverse or supplement the nominations of the executive committee could hope to succeed. The non-contents may number a considerable proportion of the whole body, and may very likely all agree to erase certain names. But they cannot, for the reason above stated, agree together upon the names to be substituted for these, and therefore their efforts are foredoomed to failure. A certain proportion of members out of the whole total will probably assent to the advice of the committee. Others will be content to testify indifference or disapproval by not voting at all. Even if no more than eighty or one hundred members return the ballot-papers in accordance with instructions, the resultant Council, though merely the nominees of the executive committee, must be declared duly elected.

That over 100 nominations have been received for the dozen places on the College Council vacant by the automatic withdrawal of one-third of its members proves that the privilege of nominating was highly appreciated. The receipt of a list of 100 names is a great opportunity for exercising an independent judgment on the part of voters who may only choose a stated number. But in this matter the nursing profession has an advantage which is possessed by few bodies. Nurses know personally, and often intimately, a very large number of matrons, sisters, and nurses. If they do not know them, they have heard about them from those who do. In going through the list there will be many familiar names, and each one who ticks off the candidates she prefers will do so for a reason.

Compare this state of things with the voter who is called on to decide out of a hundred, or even a dozen, unknown individuals. In the one case character and attainments have full weight. In the other, pure chance dictates the choice. Popular election is never, in fact, truly popular except when circumstances permit intimate knowledge of the motives, policy, and character of the persons for whom the vote is cast. This was the case in ancient Athens, where the size of the city enabled every candidate for office to be personally known to the voters, and never since has democracy borne such rich fruits. To work with people is to know them, and we have every confidence that the result of the College elections, on a democratic basis, will be to show that the opportunities of intimate association which are afforded to College members in their work one with another are being used for the common good by a discriminating selection of councillors able to guide the infant College into paths of peace and progress. Every member ought to apply her stock of information and judgment to the task of voting so that these elections may open up a new and stronger phase of union among nurses, while at the same time they may set other associations of women, perhaps of men also, to imitate a method which has demonstrated its value.

